# Real driving emissions data: Isuzu FTR850 AMT

**DOI:** 10.1016/j.dib.2022.107975

**Published:** 2022-02-22

**Authors:** Johan W. Joubert, Ruan J. Gräbe

**Affiliations:** Centre for Transport Development, Industrial & Systems Engineering, University of Pretoria, South Africa

**Keywords:** Real driving emissions, Heavy vehicle, Portable emissions measurement system, PEMS

## Abstract

RDE is becoming a necessary element of the emissions certification of automotive vehicles. Real Driving Emissions (RDE) helps to ensure that the regular operation of a car, or heavy vehicle, is still within the acceptable emissions standards while driving under normal conditions. RDE is monitored by connecting a Portable Emissions Measurement System (PEMS) to the exhaust of the tested vehicle, which measures the pollutant concentrations as the car or truck drives along a standardised route. The data described in this paper is the raw, detailed PEMS records of a heavy goods vehicle, recorded at a rate of 1Hz, over multiple trips on an urban route in South Africa. The data includes the pollutant concentrations of CO, CO${}_{2}$, NO and NO${}_{2}$, ambient conditions, and vehicle diagnostics collected from different sensors mounted to the vehicle during the field tests. We performed no additional analysis on the data. The value of the data is in allowing researchers to (a) develop and test machine learning algorithms that predict the instantaneous pollutant concentrations or (b) studying the variance of pollutant concentrations that occurs under typical driving conditions.

## Specifications Table

**Table:** 

Subject	Pollution; Automotive engineering.
Specific subject area	Real driving emissions; Portable emissions measurement system.
Type of data	Table
How data were acquired	SEMTECH®DS+ Portable Emissions Measurement System
Data format	Raw
Parameters for data collection	Predesigned routes around the City of Tshwane Metropolitan Municipality (Pretoria, South Africa) with different road grade, road types, congestion levels, all occurring approximately 1400 m above sea level.
Description of data collection	Connecting the SEMTECH®DS+ PEMS unit to the exhaust of an Isuzu FTR850 AMT truck; warming up and calibrating the unit; driving along predetermined routes. See Figs. 1 and 2 for equipment setup.
Data source location	Institution: Centre for Transport Development, Department of Industrial and Systems Engineering, University of Pretoria
	City/Town/Region: Pretoria, City of Tshwane Metropolitan Municipality, Gauteng
	Country: South Africa
	Latitude and longitude for collected samples/data: 25${}^{\circ }$45${}^{\prime }$ 10.0${}^{\prime \prime }$ S, 28${}^{\circ }$13${}^{\prime }$ 40.1${}^{\prime \prime }$E (route origin)
Data accessibility	Repository name: Mendeley
	Data identification number: 10.17632/y9pjtt5ngc
	Direct URL to data: https://doi.org/10.17632/y9pjtt5ngc

## Value of the Data

• Raw emissions concentrations and ambient and vehicle diagnostics collected at 1 Hz under realistic driving conditions. For a more detailed discussion on RDE and vehicle pollutants, see Suarez-Bertoa et al. [1].
• Valuable data to develop predictive models for real-time emissions.
• Data allows for the studying of the variance of pollutant concentrations under normal driving conditions.
• Accurate vehicle emission estimates allow for more accurate mobile source values for chemistry transport models and air quality studies.

## 1 Data Description

This data set contains a single data file, public-rrv.csv.gz, that is a compressed (using GNU zip) comma-separated value file. The first line contains a header row, and each next line represents a single observation recorded by the SEMTECH®DS+ PEMS unit. Table 1 describes each of the data fields provided in the file.

**Table 1:** Field description.

Column	Field name	Description	Unit of measure
1	date	Date and time in GMT + 2 (South African Standard Time).	
2	trip	Trip identifier, sequentially starting at 1. One trip is a single field test completing a single route.	
3	driver	Driver number, sequentially starting at 1.	
4	route	Route number; 1 for C; 2 for T; and 3 for d	
5	load	Additional load (balast) added to the vehicle.	kg

6	gps_lat	Latitude in WGS84.	decimal degrees
7	gps_lon	Longitude in WGS84.	decimal degrees
8	gps_alt	Altitude.	Metres above sea level
9	gps_speed	Vehicle speed derived from the GPS unit.	km/h

10	humidity	Ambient humidity.	%RH (relative humudity)
11	pressure	Ambient air pressure.	mbar
12	temp	Ambient temperature.	${}^{\circ }$C

13	speed_vehicle	Vehicle speed as recorded from OBDII port.	km/h
14	throttle	Absolute throttle position.	%
15	manifold_pressure	Pressure in the fuel/air mixture between the throttle and the engine.	kPa
16	manifold_temp	Temperature of the air inside the intake manifold.	${}^{\circ }$C
17	coolant_temp	Engine coolant temperature.	${}^{\circ }$C
18	fuel_flow	Instantaneous fuel flow.	g/s
19	fuel_rate	Fuel flow rate.	gal/s
20	air_fuel_ratio	Air/fuel ratio of the gas sample.${}^{★}$	
21	exh_humidity	Humidity of the exhaust.	%
22	exh_mass_flow	Exhaust mass flow rate.	kg/h
23	exh_flow_scfm	Exhaust volumetric flow rate.	SCFM (standard ft${}^{3}$)
24	exh_flow_ls	Exhaust volumetric flow rate.	l/s (referenced at 0${}^{\circ }$C)
25	exh_temp	Exhaust temperature.	${}^{\circ }$C
26	mass_flow_rate	Mass air flow rate.	g/s
27	CO2_amb_conc	Ambient concentration of CO${}_{2}$.	%
28	CO_amb_conc	Ambient concentration of CO.	%
29	NO_amb_conc	Ambient concentration of NO.	ppm (parts per million)
30	NO2_amb_conc	Ambient concentration of NO${}_{2}$.	ppm
31	O2_amb_conc	Ambient concentration of O${}_{2}$.	%
32	CO2_wet_conc	Wet concentration of CO${}_{2}$.	%
33	CO_wet_conc	Wet concentration of CO.	%
34	NO_wet_conc	Wet concentration of NO.	ppm
35	NO2_wet_conc	Wet concentration of NO${}_{2}$.	ppm
36	NOx_wet_conc	Wet concentration of NO${}_{\text{x}}$ (Nitrogen Oxides).	ppm
37	O2_wet_conc	Wet concentration of O${}_{2}$.	%
38	CO2_mass	Instantaneous mass CO${}_{2}.^{★}$	g/s
39	CO_mass	Instantaneous mass CO.${}^{★}$	g/s
40	NO_mass	Instantaneous mass NO.${}^{★}$	g/s
41	NO2_mass	Instantaneous mass NO${}_{2}.^{★}$	g/s
42	NOx_mass	Instantaneous mass NO${}_{\text{x}}.^{★}$	g/s
43	O2_mass	Instantaneous mass O${}_{2}.^{★}$	g/s
44	NO_mass_cor	Corrected instantaneous mass CO${}_{2}.^{★}$	g/s
45	NO2_mass_cor	Corrected instantaneous mass NO${}_{2}.^{★}$	g/s
46	NOx_mass_cor	Corrected instantaneous mass NO${}_{\text{x}}.^{★}$	g/s

${}^{★}$ calculated fields.

The data field are grouped by function and source. The first field is the specific time stamp of the record. Fields 2–5 provide a unique identifier for a field test trip. The trip number is just a sequential identifier that represents a unique driver completing a specific route with the vehicle. If there are additional weight loaded to the vehicle, this is reflected in field 5.

Spatial data (fields 6–9) is captured using a Garmin Global Positioning System (GPS) module integrated with the PEMS unit. A weather probe is also integrated into the unit and provides ambient readings in fields 10–12. The PEMS unit has an integrated In-vehicle Control Module (ICM) that allows the driver to record event markers (flags) during a field test. The ICM also connects to and records the vehicle’s Onboard Diagnostics (OBDII) port while driving. The vehicle diagnostics are presented in fields 13–20.

The remainder of the fields, 21–46, are all collected by the various sensors of the SEMTECH®DS+ gaseous analyser. Exhaust gasses pass through the 4-inch (±100 mm) Exhaust Flow Meter (EFM) tube, responsible for measuring the raw exhaust mass flows. The EFM operates under Bernoulli’s principle using averaging pitot tubes and employing five dual-stage, differential pressure transducers. The gas analyser unit houses the analytical devices for the gaseous measurements of CO, CO${}_{2}$, NO, and NO${}_{2}$.

## 2 Experimental Design, Materials and Methods

**Fig. 1:**
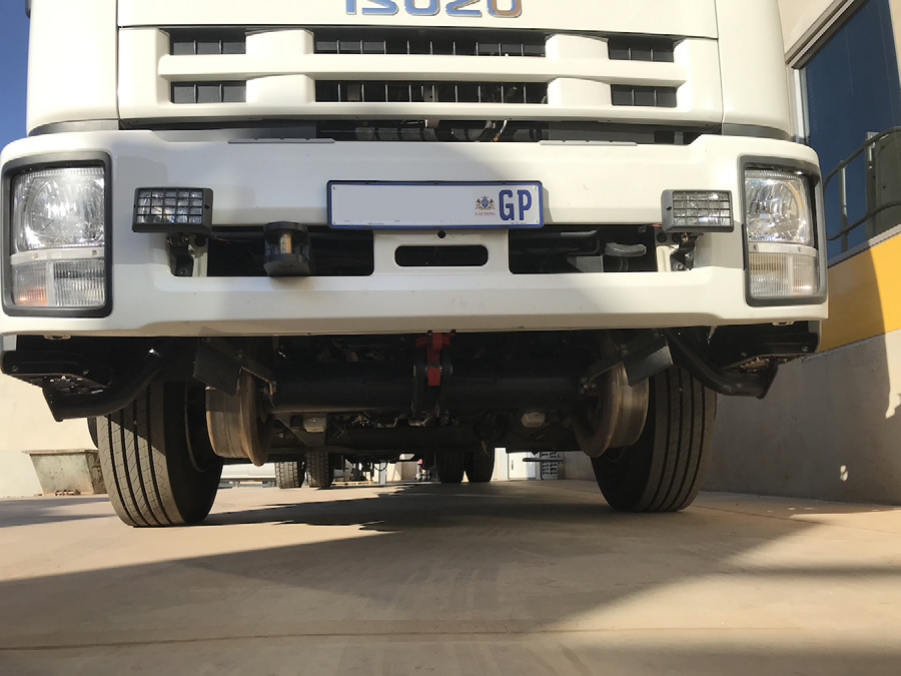
Front of the RRV showing the retracted rail subassembly.

The vehicle we use in this data set is a heavy goods research vehicle based on an Isuzu FTR850 AMT. The 7.8-litre, six-cylinder turbocharged, intercooled, common-rail diesel engine has a Euro 3 emissions rating. As a Road-Rail Vehicle (RRV), the University commissioned the truck’s customisation to operate on both rail tracks and conventional roads. The flat deck has a crane fitted, as well as a small cabin. The rail subassembly that allows the vehicle to operate on the rail and the auxiliary equipment does not directly affect the vehicle’s drive train but adds additional weight to the truck. The total weight of the vehicle, equipment and driver is ±10,740 kg with 4830 kg on the front axle and 5910 kg on the rear axle. Fig. 1 shows the rail subassembly hydraulically retracted during road use.

This specific data set only includes a single vehicle to limit the number of assignable causes affecting emission concentrations. The same vehicle is driven along a fixed set of standardised routes to allow the study of variation in pollutant concentrations while controlling for some of the variables like the vehicle itself and the standard route profile.

**Fig. 2:**
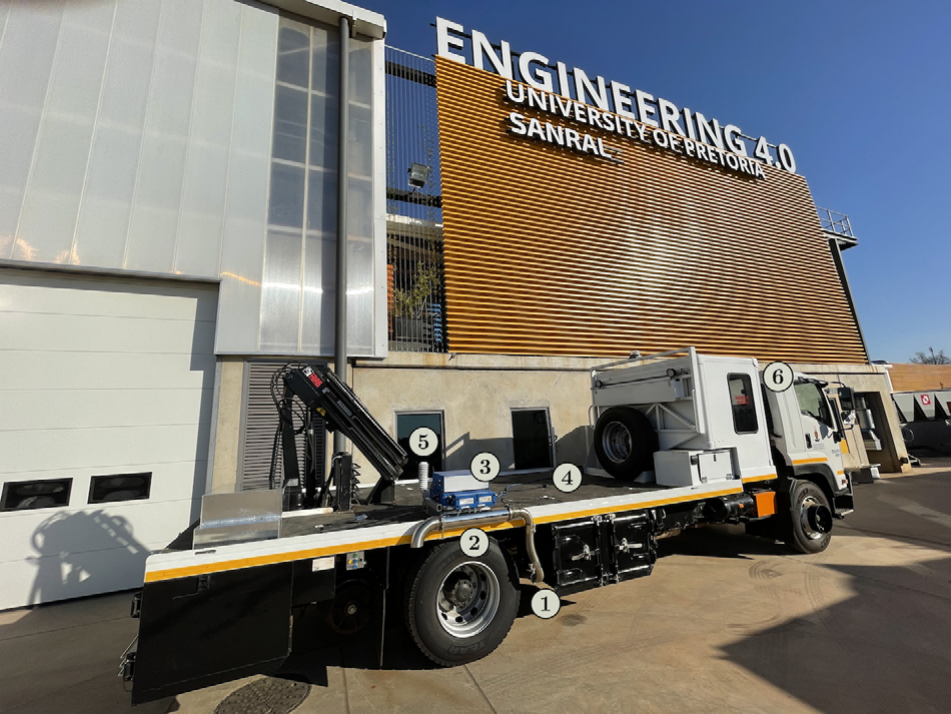
Configuration of the PEMS: ① exhaust connection; ② EFM flow tube; ③ main gaseous analyser; ④ GPS antenna; ⑤ weather probe; ⑥ ICM in the driver cab.

The SEMTECH DS+ unit is loaded and secured onto the truck’s deck, close to the exhaust. The layout of the setup on the vehicle is shown in Fig. 2. The 3-inch (±76 mm) exhaust, ①, is connected to the 4-inch EFM flow tube, ②, using a flexible stainless steel tube with a conic reducer. In this setup, the EFM connects directly to the gaseous analyser, ③. We position the GPS unit’s antenna, ④, and weather probe, ⑤, close to the centre of the deck. The ICM connects via an extended cable and is located inside the driver cab, ⑥, to connect to the truck’s OBDII port.

After completing the installation, we switch on the unit using shore power (220 V AC from the electrical grid) and allow all the modules to heat sufficiently. The monitoring of the system occurs via its built-in web server, accessed via any mobile device connected to its wireless network. The heating takes approximately 45 min. Once heated, we perform the system calibration, facilitated by the unit’s onboard software. First, we complete a zero-calibration of all sensors using ambient air and ensure that the time alignment between the different sensory modules is correct.

Next, we perform a span calibration to ensure that the unit covers the full range of pollutant concentrations expected during the field tests. The pollutants for which we complete a span calibration is CO, CO${}_{\text{2}}$, NO and NO${}_{\text{2}}$. A final leak test ensures that the sampled pollutant concentrations are not contaminated or diluted between the point of sampling in the EFM and the point of analysis in the gaseous analyser. At this point, the onboard software will give an all-clear for the pre-test procedure, and the actual field test can start.

Before the field test starts, the last step is to switch the DS+ unit over from shore power to its dedicated power source: a 13 V Lithium Iron Phosphate (LiFePO4) battery with a 108 Ah capacity. The purpose of the power source independent of the vehicle’s battery is not to place an additional burden on the truck’s alternator to charge and power the DS+, potentially affecting fuel consumption and emissions. The exposed portions of the PEMS electronics are covered with an aluminium weather box that can be seen, removed from the unit, in Fig. 2 at the back of the deck.

The (co)driver places a data marker in the field test recording, using the ICM unit. The truck is started. The route followed in a field test is one of three predetermined routes that all start and end at the Engineering 2 building on the periphery of the main Hatfield Campus of the University of Pretoria, in University Road. For the first version of the data accompanying this manuscript, only the C-shaped route (encoded as 1) reflects. Also, only a single driver (encoded as 1) has the necessary licenses and permits to operate the heavy vehicle. Future versions of the data will reflect all three routes and (potentially) multiple drivers as the field tests continue and expand.

Each route includes different road types: residential, local, secondary, primary and freeway sections and is about 60 km long and takes approximately 2 h to complete in the typical urban traffic conditions. There are also different road grades represented. After completing the trip, the (co)driver places another data marker in the field test recording. Afterwards, the research team extracts all records between two such data markers as a single trip without additional analysis. The extraction procedure uses the Sensors Inc. postprocessing software provided with the DS+ unit. We consider a trip *successful* and include it into that data set if there was no break in communication with one of the sensors during the field test. For example, we removed some trips where the connection with the truck’s OBDII port was interrupted for a few minutes without researchers noticing.

The research team tolerated only minor deviations from the route. For example, during one of the early days, there was a critical incident at the Fountains circle (25${}^{\circ }$46${}^{\prime }$ 23.8${}^{\prime \prime }$S, 28${}^{\circ }$11${}^{\prime }$ 58.1${}^{\prime \prime }$E) halfway through the C-route. The police closed a portion of the road and rerouted traffic via a detour of approximately 500 m. Later during the same day, the detour route saw vehicles crossing the median island, shortening the detour to at most 30 m. In this manuscript, we argue that such deviations are acceptable and align well with this research’s spirit: *real driving* emissions. A video of one of the trips is available online and shows the instantaneous location, traffic condition and pollutant concentrations along the route.

## Ethics Statement

The calibration gas used in the pre-test procedure includes Nitrogen dioxide (NO${}_{\text{2}}$ is an irritant but non-toxic gas, certified at 533 ppm) and carbon monoxide (CO, a toxic gas at high concentrations, certified at 4257 ppm). Authors confirm that equipment setup and calibration tests are done outdoors in well-ventilated areas without exposure to harmful quantities. The operators of the equipment and the vehicle are not exposed to toxic exhaust gasses while operating the vehicle as there is no direct exposure inside the vehicle cab.

## CRediT authorship contribution statement

**Johan W. Joubert:** Conceptualization, Funding acquisition, Methodology, Software, Investigation, Resources, Data curation, Writing – original draft. **Ruan J. Gräbe:** Formal analysis, Validation, Investigation, Data curation, Writing – review & editing.

## Declaration of Competing Interest

The authors declare that they have no known competing financial interests or personal relationships that could have appeared to influence the work reported in this paper.
